# Exposure to depleted uranium does not alter the co-expression of HER-2/neu and p53 in breast cancer patients

**DOI:** 10.1186/1756-0500-4-87

**Published:** 2011-03-29

**Authors:** Mais M Al-Mumen, Asad A Al-Janabi, Alaa S Jumaa, Kaswer M Al-Toriahi, Akeel A Yasseen

**Affiliations:** 1 Department of Pathology and Forensic Medicine, Faculty of Medicine, Kufa, University, Kufa, P.O. Box 18, Iraq

## Abstract

**Background:**

Amongst the extensive literature on immunohistochemical profile of breast cancer, very little is found on populations exposed to a potential risk factor such as depleted uranium. This study looked at the immunohistochemical expression of HER-2/neu (c-erbB2) and p53 in different histological types of breast cancer found in the middle Euphrates region of Iraq, where the population has been exposed to high levels of depleted uranium.

**Findings:**

The present investigation was performed over a period starting from September 2008 to April 2009. Formalin-fixed, paraffin-embedded blocks from 70 patients with breast cancer (62 ductal and 8 lobular carcinoma) were included in this study. A group of 25 patients with fibroadenoma was included as a comparative group, and 20 samples of normal breast tissue sections were used as controls. Labeled streptavidin-biotin (LSAB+) complex method was employed for immunohistochemical detection of HER-2/neu and p53.

The detection rate of HER-2/neu and p53 immunohistochemical expression were 47.14% and 35.71% respectively in malignant tumors; expression was negative in the comparative and control groups (p < 0.05).

HER-2/neu immunostaining was significantly associated with histological type, tumor size, nodal involvement, and recurrence of breast carcinoma (*p *< 0.05), p53 immunostaining was significantly associated with tumor size, nodal involvement and recurrence of breast cancer (*p *< 0.05). There was greater immunoexpression of HER-2/neu in breast cancer in this population, compared with findings in other populations.

Both biomarkers were positively correlated with each other. Furthermore, all the cases that co-expressed both HER-2/neu and p53 showed the most unfavorable biopathological profile.

**Conclusion:**

P53 and HER-2/neu over-expression play an important role in pathogenesis of breast carcinoma. The findings indicate that in regions exposed to high levels of depleted uranium, although p53 and HER-2/neu overexpression are both high, correlation of their expression with age, grade, tumor size, recurrence and lymph node involvement is similar to studies that have been conducted on populations not exposed to depleted uranium. HER-2/neu expression in breast cancer was higher in this population, compared with results on non-exposed populations.

## Introduction

Breast carcinoma constitutes around one quarter of all cancers, making it the most common cancer in females [1], it is a heterogeneous disease with high individual variability as far as response to treatment is concerned [2]. Despite the increasing incidence rates of breast cancer, the morbidity and mortality rates are beginning to fall. This decrease may reflect improvements in methods of diagnosis and treatment [3]. Several molecular markers that are important in the clinical aspect of malignancies especially in breast cancer have been detected [4]. Biological markers capable of predicting responsiveness to therapy would be of great help to physicians aiming to select the most appropriate treatment for each patient [5]. HER2/neu and p53 (both genes map to chromosome 17) are known biomarkers of breast cancer. HER2/neu is a member of the epidermal growth factor receptor (EGFR) family. Activation of the HER2/neu gene results in synthesis of 185kD transmembrane protein, whose intracellular domain possesses tyrosine kinase activity and through phosphorylation induction leads to tumor cell proliferation [6]. HER2/neu amplification or over- expression has been shown to be associated with higher grades of tumor and poorer prognosis [6,7]. P53 is involved in regulating cell proliferation, including apoptosis, and in promoting chromosomal stability. It has been demonstrated that some percentage of breast cancers with mutations in p53 tumor suppressor gene are associated with clinical aggressiveness [8-12]. The prognostic and predictive value of HER2 and p53 biomarkers has been the subject of many investigations [10,11], though the significance of their expression in cancer prognosis requires further study.

With respect to hormone receptors status (estrogen (ER) and progesterone (PR)), Breast cancer is considered as a hormone - dependent cancer. In published literature, approximately 50% of tumors are ER+ PR+; 25% ER- PR-; 20% ER+ PR- and 5% are ER-PR+ [13,14]. Indeed, the estrogen receptor (ER) and the progesterone receptor (PR) have been widely accepted as established parameter in the management of patients with primary breast cancer along with HER-2/neu and P53 status as prognostic markers. Her-2/neu shows over- expression in high grade cancer and displays lower responsiveness to hormone receptors modulators. Hormone receptors positivity also correlate with absence of p53 mutation [15] and inversely with the presence of epidermal growth factor receptor [16].

On the other hand, Uranium exposure risk has been assessed in terms of radiation exposure. Human exposure to DU can occur through various routes including inhalation of DU particles, ingestion of DU-contaminated drinking water, soil, food or penetration of the body by bullets or shrapnel. It has been estimated that that 371 tones of DU were deposited in Iraqi Soil during the Desert Storm alone [17]. Recent literature includes much experimental evidence of adverse effects of DU including: altered gene expression in vitro [18], DNA strand breakage [19], carcinogenic mutation of human bronchial tissue [20] and genomic instability of human osteoblasts [21,22]. DU also produces increased urinary mutagenicity using the Ames test in rats [23]. Indeed, exposure to radiation leads to mutations in components of signaling pathways that control cell growth. Ultimately, these changes drive tumorigenesis through the coordinated phosphorylation of proteins, cell-cycle progression and metabolism, and transcription factors that regulate the expression of genes involved in these processes [24,25]. The mutations that cause cancer is produced by complex interactions between environmental and inherited factors. It is unlikely that a single specific abnormality causes all breast cancer. Defective P53 tumor suppressor gene, could allow abnormal cells to proliferate, resulting ultimately in cancer. As many as 50% of all human tumors are associated with P53 mutations [26]. HER-2 is encoded by the erbB-2 gene, the human homologue of the rat proto-oncogen neu [27]. Damage to either type of genes (tumor - suppressor gene and proto-oncogene) due to exposure to environmental factors (e.g. DU) can results in uncontrolled division of cells. This uncontrolled division forms tumors.

The present study is the first to look at the co-expression of these biomarkers in breast tissue samples from Iraqi women of the middle Euphrates area where the population was exposed to high levels of depleted uranium following the Gulf War. The results of this work are compared to findings on co-expression of these biomarkers in studies on breast cancer from other parts of the world, where depleted uranium exposure is not a risk.

## Methods

Approval for the study was granted by the Iraqi Ministry of Higher Education and Scientific Research Ethics Committee and followed the Tents of Declaration of Helsinki. The Authors wrote to the patients asking if they would be willing to donate their tissues for the project. Families of individuals were also contacted. The samples were taken from consenting individuals and their families.

Seventy specimens of formalin-fixed, paraffin embedded breast cancer tissue, collected from breast cancer patients over a period from September 2008 to April 2009 were included in this study. All cases were referred to Kufa School of Medicine Teaching Hospital for histopathological evaluation from different parts of the middle Euphrates region of Iraq. The age range of patients was 22 to 70 years, with a mean age of 46.9 years. A group of 25 patients with benign breast lesions (fibroadenoma) was included as a comparative group and 20 normal breast tissue sections were included as controls. Confirmation of histopathological diagnosis and grading of tumors were carried out after reviewing all slides before proceeding further to the immunohistochemical analysis. Tissue sections with a thickness of 4 μm were taken from the formalin-fixed, paraffin embedded blocks for immunohistochemistry. Labeled streptavidin-biotin (LSAB+) method was employed for immunohistochemical detection of HER-2/neu and p53 using Polyclonal Rabbit Anti- Human c-erbB-2 Oncoprotein, Code No. 0485, Dako Denmark A/S Produktionsvej 42 DK-2600 Glostrup and Monoclonal Mouse Anti-Human p53 Protein, Ready-To-Use, DAKO, Clone DO-7, Code N1581, Inc. 6392, CA 93013 USA. The intensity of HER-2/neu cell membrane stain was classified into score 0 (completely negative), score 1+ (negative; just perceptible staining of the membrane in > 10% of the malignant cells), score 2+ (moderate staining of the partial membrane in > 10% of the malignant cells) and score 3+ (strong circumferential staining of the entire membrane creating a fish-net pattern in > 10% of the malignant cells) [11]. The intensity of p53 nuclear stain was classified into score 0 (negative), score 1+ (weak or mild staining, with 5-10% tumor cells staining positive), score 2+ (moderate staining with less than 25% of tumor cells staining positive), score 3+ (strong staining, with 25-50% of tumor cells staining positive) and score 4+ (highly strong staining with over 50% of tumor cells staining positive)[12]. All biopsies were classified into three grades: Grade I, Grade II and Grade III, according to the modified Bloom Richardson Grading System [28]. The results were statistically evaluated with a Chi-squared test (at a significant level of p <0.05) and correlation-regression analysis (at a significance level of R = 0.3) using SSPS software.

## Results

HER-2/neu and p53 immunoexpressions were positive in 47.14% and 35.71% of breast cancer cases, respectively, and negative in all sections of the normal and benign breast tissues. The differences between these groups were statistically significant (*p *< 0.05) (Table 1). HER-2/neu over-expression was detected in 51.61% of ductal carcinomas cases and in 12.5% of invasive lobular carcinoma. The difference was statistically significant (*p *< 0.05).

**Table 1:** Immunoexpression of HER-2/neu and p53 in relation to clinicopathological parameters of breast carcinoma

Parameters	Total number of patients No. %	HER-2 overexpression		P53 immunoexpression	
		Positive No. %	Negative No. %	Positive No. %	Negative No. %
**Type of breast tissue**					
Normal	20 (17.39)	0 (0)	20 (100)	0 (0)	20 (100)
Benign (fibroadenoma)	25 (21.74)	0 (0)	25 (100)	0 (0)	25 (100)
Malignant	70 (60.87)	33 (47.14)	37 (52.86)	25 (35.71)	45 (64.29)
	**P < 0.05**			**P < 0.05**	
**Histological type**					
Lobular carcinomas	8 (11.43)	1 (12.50)	7 (87.50)	2 (25)	6 (75)
Ductal carcinomas including:	62 (88.57)	32 (51.61)	30 (48.39)	23 (37.10)	39 (62.90)
	**P < 0.05**			**P > 0.05**	
Pure IDC	47 (67.14)	19 (40.43)	28 (59.57)	18 (38.30)	29 (61.70)
IDC + DCIS	9 (12.86)	7 (77.68)	2 (22.22)	2 (22.22)	7 (77.78)
IDC+ Paget's	2 (2.86)	2 (100)	0 (0)	1 (50)	1 (50)
DCIS + Paget's	1 (1.44)	1 (100)	0 (0)	1 (100)	0 (0)
Pure DCIS	3 (4.29)	3 (100)	0 (0)	1 (33.33)	2 (66.67)
	**P < 0.05**			**P > 0.05**	
**Tumor grade**					
Well-moderately differentiated (I and II)	14 (20)	7 (50)	7 (50)	2 (14.29)	12 (85.71)
Poorly differentiated (III)	56 (80)	26 (46.43)	30 (53.57)	23 (41.07)	33 (58.93)
	**P > 0.05, R > 0.3**			**P > 0.05**,	**R > 0.3**
**Tumor size**					
Tis	4 (5.71)	4 (100)	0 (0)	2 (50)	2 (50)
T1 (≤ 2 cm)	7 (10)	2 (28.57)	5 (71.43)	2 (28.57)	5 (71.43)
T2 (2 > -5 cm)	38 (54.29)	13 (34.21)	25 (65.79)	6 (15.79)	32 (84.21)
T3 (> 5 cm)	19 (27.14)	13 (68.42)	6 (31.58)	14 (73.68)	5 (26.32)
T4 (anyT+other)	2 (2.86)	1 (50)	1 (50)	1 (50)	1 (50)
	**P < 0.05**	**R < 0.3**		**P < 0.05**	**R > 0.3**
**Axillary lymph nodes1**					
Negative	27 (48.21)	6 (22.22)	2 (77.78)	7 (25.93)	20 (74.07)
Positive	29 (51.79)	19 (65.52)	10 (34.48)	17 (58.62)	12 (41.38)
	**P < 0.05**			**P < 0.05**	
**Age of the patient**					
< 35 years	19 (27.14)	12 (63.16)	7 (36.84)	8 (42.11)	11 (57.89)
> 35years	51 (72.86)	21 (41.18)	30 (58.82)	17 (33.33)	34 (66.67)
	**P > 0.05**			**P > 0.05**	
**Tumor recurrence**					
Primary	51 (72.86)	17 (33.33)	34 (66.67)	13 (25.49)	38 (74.51)
Recurrent	19 (27.14)	16 (84.21)	3 (15.79)	12 (63.16)	7 (36.84)
	**P < 0.05**			**P < 0.05**	

**Figure 1:**
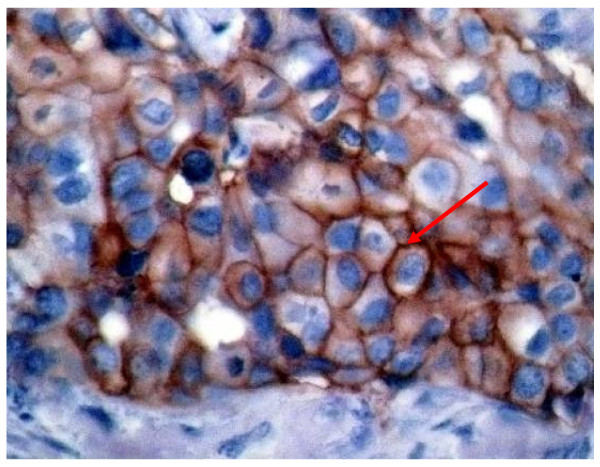
**Invasive ductal carcinoma, showing strong membranous staining of Her2/neu, score 2+ (arrowed) (40×)**.

**Figure 2:**
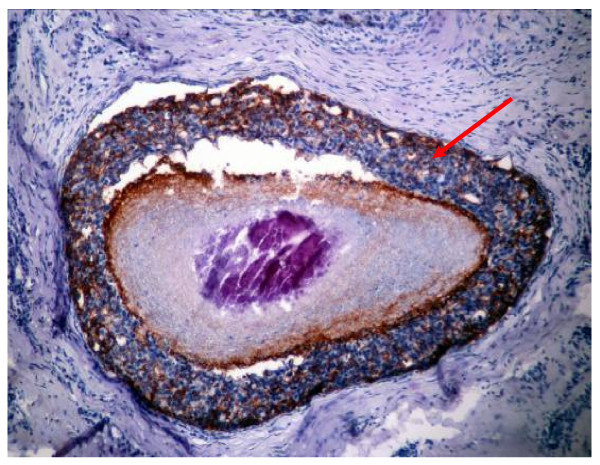
**In situ ductal carcinoma of comedo type, showing strong membranous staining of Her2/neu, score 2+ (arrowed) (10×)**.

As grouped in Table 1, over- expression of HER-2/neu was detected in only 40.43% of those with pure invasive ductal carcinomas (Figure 1), in comparison with 77.68% of invasive ductal carcinoma with an *in situ *comedo component (DCIS), in 100% of invasive ductal carcinoma with overlying Paget's disease and in 100% of purely DCIS (Figure 2) and that with overlying Paget's disease. The differences were statistically significant (p < 0.05).

HER-2/neu overexpression was detected in 50% of both grade I and II (well-moderately differentiated breast cancer) as compared to 46.43% of Grade III (poorly differentiated breast cancer). Statistically, no significant difference exists (p > 0.05). Furthermore, a positive HER-2/neu overexpression was detected in all (100%) of the Tis size breast tumors, in 28.57% of T1 size, 34.21% of T2, 68.42% of T3 and in 50% of T4. Table 1 shows that there is a high detection rate of HER-2 overexpression in recurrent breast cancer in comparison with primary lesions (84.21% versus 33.33%)(p < 0.05), and in the presence (positive) or absence (negative) of axillary lymph nodes (p < 0.05) (65.52% versus 22.22%), a detection rate showed no correlation with patients age.

**Figure 3:**
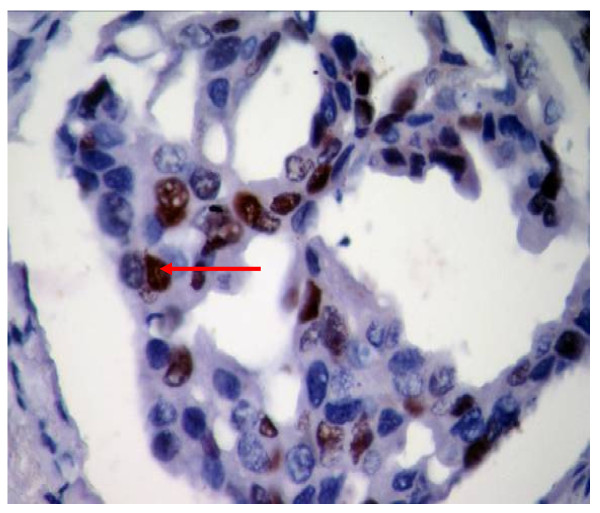
**Invasive ductal carcinoma showing strong nuclear staining of P53, score 2+ (arrowed) (40×)**.

**Figure 4:**
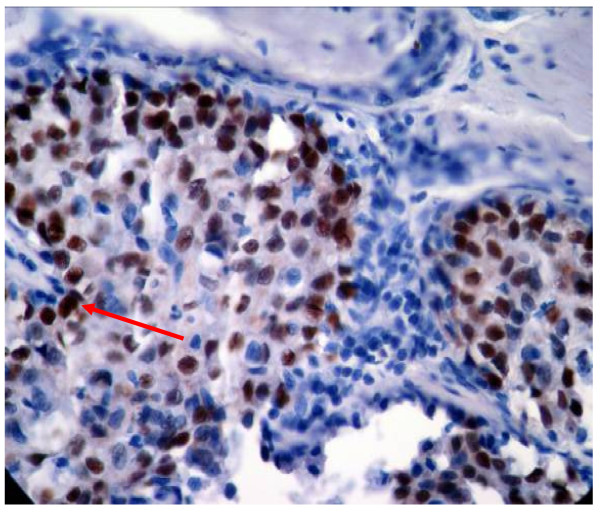
**In situ ductal carcinoma showing strong nuclear staining of P53, score 3+ (arrowed) (20×)**.

In the present study, p53 immunoexpression was detected in 37.10% of the ductal carcinomas cases and in 25% of invasive lobular carcinoma only (Table 1). P53 Immunoexpression was also detected in 38.30% of those tumors with pure invasive ductal carcinomas (Figure 3), in 22.22% of invasive ductal carcinoma with an *in situ *comedo component (DCIS), in 50% of invasive ductal carcinoma with overlying Paget's disease, in 33.33% for purely DCIS (Figure 4) and in 100% of those with overlying Paget's disease, in all these cases the incidence is significantly different from that found in control (p < 0.05). On the other hand, p53 immunoexpression was detected in 14.29% of both grade I and II (well-moderately differentiated breast cancer) as compared to 41.07% of Grade III (poorly differentiated breast cancer). It is obvious that p53 immunoexpression was highly correlated with the grade of tumor (R = 0.9) though no statistical significant difference was found (P > 0.05). A positive p53 immunoexpression was detected in 50% of Tis size of tumor, in 28.57% of T1, in 15.79% of T2, in 73.68% of T3 and in 50% of T4 size of tumor. A higher detection rates of p53 immunoexpression was found in recurrent breast cancer patients compared with the primary lesions (63.16% versus 25.49%) (p < 0.05) and in the presence (Positive) or absence (negative) of auxiliary lymph nodes (58.62% versus 25.93%). Again, there was a high detection rate in both age groups but no correlation with age was found. Both biomarkers are positively correlated with each others with respect to most clinicopathological parameters(R = 0.9). The percentage of the cases coexpressing both tumor markers is 18.57% (Table 2). All these cases showed the worst biopathological profile. The cases that co-expressed both biomarkers were found in 19.35% of ductal carcinoma, in 21.43% of poorly differentiated (grade III) tumor, in 50% of T4 tumor size, in 43.49% of those with positive axillary lymph node and in 47.37% of those that showed recurrence tumors. Furthermore, the co-expression of both biomarkers was significantly correlated with tumor grade and decreasing patients age (R > 0.3) (Table 2).

**Table 2:** Coexpression of HER-2/neu and p53 in relation to clinicopathological parameters of breast carcinoma

Parameters	Both HER-2/neu and p53 positive	Only HER-2/neu positive	Only p53 positive	both HER-2/neu and p53 negative	Total
**Histological type**					
**Ductal Carcinoma**	12 (19.35%)	20 (32.25%)	11 (17.45%)	19 (30.65%)	**62** **(88.57%)**
**Lobular Carcinoma**	1 (12.50%)	0 (0%)	1 (12.50%)	6 (75%)	**8** **(11.43%)**
	**R > 0.3**				
**Tumor grade**					
**Well-moderately differentiated** **(I and II)**	1 (7.14%)	6 (42.86%)	1 (7.14%)	6 (42.86%)	**2** **(2.86%)**
**Poorly differentiated (III)**	12 (21.43%)	14 (25%)	11 (19.64%)	19 (33.93%)	**56** **(80%)**
	**R < 0.3**				
**Tumor size**					
**Tis**	2 (50%)	2 (50%)	0 (0%)	0 (0%)	**4** **(5.71%)**
**T1**	0 (0%)	2 (28.57%)	2 (28.57%)	3 (42.86%)	**7** **(10%)**
**T2**	2 (5.26%)	11 (28.94%)	4 (10.53%)	21 (55.27%)	**38** **(54.29%)**
**T3**	8 (42.12%)	5 (26.31%)	6 (31.57%)	0 (0%)	**19** **(27.14%)**
**T4**	1 (50%)	0 (0%)	0 (0%)	1 (50%)	**2** **(2.86%)**
	**R > 0.3**				
**Axillary lymph nodes**					
**Node +ve** **breast cancer**	10 (34.49%)	9 (31.03%)	7 (24.14%)	3 (10.34%)	**29** **(51.79%)**
**Node-ve** **breast cancer**	2 (7.41%)	4 (14.81%)	5 (18.52%)	16 (59.26%)	**27** **(48.21%)**
	**R < 0.3**				
**Age of the patient**					
**< 35 years**	8 (4211%)	4 (21.05%)	0 (0%)	7 (36.84%)	**19** **(27.14%)**
**> 35years**	5 (9.81%)	16 (31.37%)	12 (23.53%)	18 (35.29%)	**51** **(72.86%)**
	**R > -0.3**				
**Tumor recurrence**					
**Primary**	4 (7.84%)	13 (25.49%)	9 (17.65%)	25 (49.02%)	**51** **(72.86%)**
**Recurrent**	9 (47.37%)	7 (36.84%)	3 (15.79%)	0 (0%)	**19** **(27.14%)**
**Total**	**13** **(18.57%)**	**20** **(28.57%)**	**12** **(17.14%)**	**25** **(35.71%)**	**70** **(100%)**
	**R > 0.3**				

## Discussion

Depleted uranium levels were estimated to be around 320-800 tons in the aftermath of the first Gulf war in 1991 with further comparable levels occurring in 2003. Since the targets were always in heavily populated areas in the middle and south of Iraq, the extent of population exposure has been extensive [29]. It has not been possible to obtain accurate measures of exposure because of official restrictions and the current political situation. Whilst there is plenty of anecdotal evidence for increases in the incidence of malignancies, infertility and congenital malformations, there is a paucity of studies to investigate these conditions on Iraqi populations. This has been partly because of difficulties in obtaining samples compounded by the destruction of laboratory facilities and the lack of scientific and medical personnel. This study is the first to study breast cancer in an Iraqi population that had been exposed to high levels of depleted uranium and examine how the immunoexpression of HER-/neu and p53 compared with tumor grade and size.

In the present investigation the percentage of immunoexpression in malignant breast lesions ranges from 12.5% to 100% for HER-2/neu and from 22.22%-100% for p53. This study has found that HER-2/neu was overexpressed in 47.14% out of 70 breast cancer cases, a result that is higher than those reported elsewhere [30-32]. This may reflect the variant genetic make-up in different ethnic groups or may be an effe**c**t of environmental damaging agents.

Furthermore, previous work that had been carried out in the same area showed relatively higher HER-2/neu over- expression (67.8%) compared to the present investigation, though there was no significant difference was found between the two studies, the differences between the two can possibly be attributed to either the differences in the sample sizes, the period of samples collection or the sensitivity of the kit that had been used [33].

The immunoexpression of HER-2/neu in ductal carcinomas was significantly greater than that in infiltrating lobular carcinomas. The rate of expression of this biological marker was similar in both Paget cells and in the underlying intraductal and/or ductal carcinoma cells.

The current study showed that 100% of pure DCIS and 77.78% of DCIS with invasive component were HER2/neu positive. These results are consistent with previous findings that indicated a role of HER-2/neu in initiation rather than in progression of ductal carcinomas and suggested that this biomarker decreases as individual tumor cells evolve from in situ to increasingly invasive lesions [32]. HER-2/neu overexpression was seen in all cases which showed either invasive ductal carcinoma with Paget's disease or ductal carcinoma in situ with overlying Paget's disease components.

The proportions of purely invasive ductal carcinomas, purely DCIS and DCIS with invasive component that were p53 positive were similar to results found in other studies[34]. Paget's disease showed p53 expression in 50% of cases with an invasive component and in all cases which had an intra-ductal growth pattern.

Although a positive correlation between detection of HER-2/neu and p53 biomarkers with the grade of tumor was observed, no statistically significant difference was seen between these grades when compared for both biomarkers. This suggests that as the tumor advances other biological changes may occur that reduce the requirement for continued biomarker signaling. It is also possible that, when gene alterations occur in breast cancer, high proliferation rates are found irrespective of the presence of invasion and that other molecular alterations are involved in the development of breast cancer [35], Accordingly, the degree of differentiation does not contribute to the increase of the expression of both markers, though it may reflect the possible role of other pathways by which the tumor is advancing independently from the increase in signaling pathways of both HER2/neu and p53 genes. Thus, introducing a new line of treatment which include a genetic modulation of the signaling pathway may alter the prognosis of breast cancer patients which clearly requires further attention in future research and medical follow up. The detection rate of p53 increased with size of tumor and there was a significant difference among the various tumor sizes (p < 0.05). This observation is consistent with previous investigations [36-39].

There was a significantly higher HER-2/neu and p53 immunoexpression in recurrent breast cancer patients compared with the primary lesions (p < 0.05). This is comparable with findings in previous studies [34-37] as is the strong correlation between HER-2/neu and p53 co-expression and grade, lymph node and tumor recurrence found in this study[40-43]. However, dissenting results that did not find a correlation between HER-2/neu and p53 co-expression and other prognostic parameter have also been reported [44].

The present work confirms previous findings that combined alteration in the expression of HER-2/neu and p53, are linked to accelerated tumor progression and a poor prognosis [45]. Other studies, however, have suggested that tumor aggressiveness, associated with elevated expression of either protein, is not increased by the alteration of a second protein involved in the same signal transduction pathway [46].

## Conclusion

In conclusion, the positive expression of these biomarkers is associated with biologically aggressive tumors and poor prognostic profile. Although the samples were taken from an area where the exposure to depleted uranium is a risk, the incidence of co-expression of both p53 and HER-2/neu markers does not differ from similar cancer samples in areas that have not been exposed to depleted uranium, though, the greater immunoexpression of Her-2/neu in breast cancer in this population with risk for DU exposure, compared with findings on other populations not at risk, requires further investigation as it may reflect the possible role of DU in the induction or acceleration of network signaling between different Her-2 receptors. New lines of treatment which includes genetic modulation of the signaling pathway of both genes should be considered in patients' medical follow up. Unfortunately for DU, knowledge of the exposure time, dose absorbed, route, length of exposure and its health consequences on the Iraqi population is still lacking. This is chiefly due to restricted access of scientists required to conduct such study and should form the basis for future investigations.

## List of abbreviations

LSAB+: Labeled Streptavidin-biotin; EGFR: epidermal growth factor receptor; ER: estrogen; PR: progesterone; DU: depleted uranium; DCIS: ductal carcinoma in situ.

## Competing interests

The authors declare that they have no competing interests.

## Authors' contributions

MMM carried out the immunohistochemical analysis and interpretation of the data, AAA participated in the design of the study, carried out the histopathological examination and helped to draft the manuscript, ASJ performed the statistical analysis, KST participated in the histopathological examination, AAY shared the design of the study and drafted the manuscript. All authors read and approved the final version of the manuscript.
